# Systems genetics identifies miRNA-mediated regulation of host response in COVID-19

**DOI:** 10.1186/s40246-023-00494-4

**Published:** 2023-06-12

**Authors:** T. Gjorgjieva, A. Chaloemtoem, T. Shahin, O. Bayaraa, M. M. Dieng, M. Alshaikh, M. Abdalbaqi, J. Del Monte, G. Begum, C. Leonor, V. Manikandan, N. Drou, M. Arshad, M. Arnoux, N. Kumar, A. Jabari, A. Abdulle, G. ElGhazali, R. Ali, S. Y. Shaheen, J. Abdalla, F. Piano, K. C. Gunsalus, H. Daggag, H. Al Nahdi, H. Abuzeid, Y. Idaghdour

**Affiliations:** 1 Biology Program, Division of Science, New York University Abu Dhabi, Abu Dhabi, United Arab Emirates; 2 Public Health Research Center, New York University Abu Dhabi, Abu Dhabi, United Arab Emirates; 3 Center for Genomics and Systems Biology, NYU Abu Dhabi, Abu Dhabi, United Arab Emirates; 4 Seha (Abu Dhabi Health Services Company), Abu Dhabi, United Arab Emirates; 5 Sheikh Khalifa Medical City-Union 71 PureHealth, Abu Dhabi, United Arab Emirates; 6 Department of Medical Microbiology and Immunology, College of Medicine and Health Sciences, United Arab Emirates University, Al Ain, United Arab Emirates

**Keywords:** COVID-19, MicroRNA, Host immune response, Transcriptomics, eQTL, Host genetics, Multi-omics, Post-transcriptional regulation

## Abstract

**Background:**

Individuals infected with SARS-CoV-2 vary greatly in their disease severity, ranging from asymptomatic infection to severe disease. The regulation of gene expression is an important mechanism in the host immune response and can modulate the outcome of the disease. miRNAs play important roles in post-transcriptional regulation with consequences on downstream molecular and cellular host immune response processes. The nature and magnitude of miRNA perturbations associated with blood phenotypes and intensive care unit (ICU) admission in COVID-19 are poorly understood.

**Results:**

We combined multi-omics profiling—genotyping, miRNA and RNA expression, measured at the time of hospital admission soon after the onset of COVID-19 symptoms—with phenotypes from electronic health records to understand how miRNA expression contributes to variation in disease severity in a diverse cohort of 259 unvaccinated patients in Abu Dhabi, United Arab Emirates. We analyzed 62 clinical variables and expression levels of 632 miRNAs measured at admission and identified 97 miRNAs associated with 8 blood phenotypes significantly associated with later ICU admission. Integrative miRNA-mRNA cross-correlation analysis identified multiple miRNA-mRNA-blood endophenotype associations and revealed the effect of miR-143-3p on neutrophil count mediated by the expression of its target gene BCL2. We report 168 significant *cis*-miRNA expression quantitative trait loci, 57 of which implicate miRNAs associated with either ICU admission or a blood endophenotype.

**Conclusions:**

This systems genetics study has given rise to a genomic picture of the architecture of whole blood miRNAs in unvaccinated COVID-19 patients and pinpoints post-transcriptional regulation as a potential mechanism that impacts blood traits underlying COVID-19 severity. The results also highlight the impact of host genetic regulatory control of miRNA expression in early stages of COVID-19 disease.

**Supplementary Information:**

The online version contains supplementary material available at 10.1186/s40246-023-00494-4.

## Introduction

The three years since the emergence of SARS-CoV-2 have brought unprecedented progress in our scientific understanding of the SARS-CoV-2 infection and COVID-19 disease. However, one overarching question remains: why do individuals infected with SARS-CoV-2 vary in their clinical symptomatology, from asymptomatic infection to severe, and oftentimes, lethal disease [1, 2]? The answer to this complex question lies in layers of genetic, biological, environmental, and social factors. Previous studies have found that both men and older patients, as well as those with underlying medical conditions such as diabetes, hypertension and obesity are at a higher risk for severe disease, requirement of intensive care and death [3-6]. Studies have also identified a number of blood phenotypes associated with severe disease, including elevated levels of D-dimers, C-reactive protein (CRP), neutrophil-to-lymphocyte ratio (NLR), Interleukin 6 (IL-6), IL-10, lactate dehydrogenase (LDH), procalcitonin and albumin [7-14]. Neutrophils have been found to play a critical role in the pathophysiology of COVID-19 [15-17], with activation of circulating neutrophils—as observed in transcriptomic data—pinpointed as a predictor of clinical illness in COVID-19 [18, 19]. Genetic variation has also been shown to influence COVID-19 susceptibility, severity and clinical outcomes [20, 21]. While there has been extensive research to unpack the different sources of variation that influence COVID-19 disease severity, only a few studies to date have focused on the potential roles of human-encoded microRNA (miRNA). 

miRNAs are a class of small, non-coding RNAs that regulate gene expression by binding to complementary mRNA transcripts to either block translation or mark the target mRNA for degradation [22, 23]. miRNAs can regulate both neighboring or distal genes; one miRNA can regulate either one or multiple genes; and multiple miRNAs can target the same gene in either a synergistic or antagonistic manner [24]. Since regulated miRNA expression is crucial for the differentiation, activation and survival of immune cells [25], dysregulated miRNA expression can be indicative of aberrant immune function, and has been implicated in numerous diseases including cancers, inflammatory disorders and malaria [26-28]. miRNA expression is also influenced by host genetics, with a few studies describing genetic variation associated with miRNA expression in healthy donors and disease contexts like malaria and cancer [29-32].

Despite the contributions of miRNAs to immune function, our understanding of the roles of miRNAs in response to SARS-CoV-2 is still in its nascency. There are a number of studies (reviewed in Geraylow et al. [33]) that have identified aberrant miRNA expression during COVID-19 disease progression. Farr and colleagues reported the differential expression of 55 miRNAs between COVID-19 patients during the early stage of disease and healthy donors matched for age and gender [34]; Fernández-Pato and colleagues identified 200 differentially expressed miRNAs between COVID-19 patients and healthy controls which were also correlated with proinflammatory cytokines such as IL-6, IL-12, IP-10, and TNFɑ [35]; Pinacchio and colleagues highlighted increased levels of miR-122a and miR-146a in the serum of COVID-19 patients compared to controls, and reported a negative correlation between miR-146a and Interferon alpha-inducible protein 27 (IFI-27) [36]; de Gonzalo-Calvo et al. identified 10 miRNAs that were dysregulated in hospitalized patients admitted to the intensive care unit (ICU), compared to patients that did not require ICU care, reported correlations between miRNA levels and length of ICU stay, and found that the expression of miR-192-5p and miR-323a-3p differentiated ICU non-survivors from survivors [37]; Li and colleagues used mendelian randomization to pinpoint two miRNAs (hsa-miR-30a-3p and hsa-miR-139-5p) as potentially causal for COVID-19 severity [38]; and early in the pandemic, Kim and colleagues identified five miRNAs (hsa-miR-15b-5p, hsa-miR-195-5p, hsa-miR-221-3p, hsa-miR-140-3p, and hsa-miR-422a) predicted to commonly bind the SARS-CoV, MERS-CoV and SARS-CoV-2 viruses, and showed that they were differentially expressed in hamster lung tissues before and after SARS-CoV-2 infection [39]. Importantly, many of the miRNAs highlighted across these studies were shown to be enriched in inflammatory and antiviral immune response pathways [33]. Another set of studies have focused on uncovering the mechanisms behind miRNA regulation. Latini and colleagues showed a functional role for hsa-let7b-5p in modulating levels of ACE2 and DPP4—two receptors that play an important role in the onset and progression of COVID-19 disease—and established that low expression of this miRNA was associated with ACE2 and DPP4 overexpression in naso-oropharyngeal swabs in COVID-19 patients [40]. Meanwhile, seeking to better understand the mechanism behind neurological symptoms in COVID-19, Trampuž and colleagues highlighted 98 miRNAs that have been implicated in both COVID-19 and one of five neurological disorders [41]. Together, these studies implicate miRNAs in the human immune response to COVID-19 infection; however, most of them only included patient populations from Australia, Europe and North America, and none examined the effect of genome-wide genetic variation on host miRNA expression during SARS-CoV-2 infection.

In this study, we generated and analyzed a multi-omics dataset—genotypes, miRNA and mRNA expression—and phenotypes derived from electronic health records (EHRs) to understand the genetic and biological underpinnings of ICU admission and its associated blood phenotypes for a diverse group of 259 unvaccinated COVID-19 patients in Abu Dhabi, the United Arab Emirates (UAE). This systems genetic approach revealed miRNAs associated with blood traits underlying COVID-19 disease severity and progression and provide evidence for the role of post-transcriptional regulation of neutrophils in COVID-19. We also report the impact of host genetic regulatory variation on miRNA expression traits supporting the hypothesis that severity of COVID-19 is under host genetic control of post-transcriptional events in circulating immune cells.

## Results

### Systems genetics to study early stages of COVID-19 in a diverse unvaccinated cohort

**Fig. 1:**
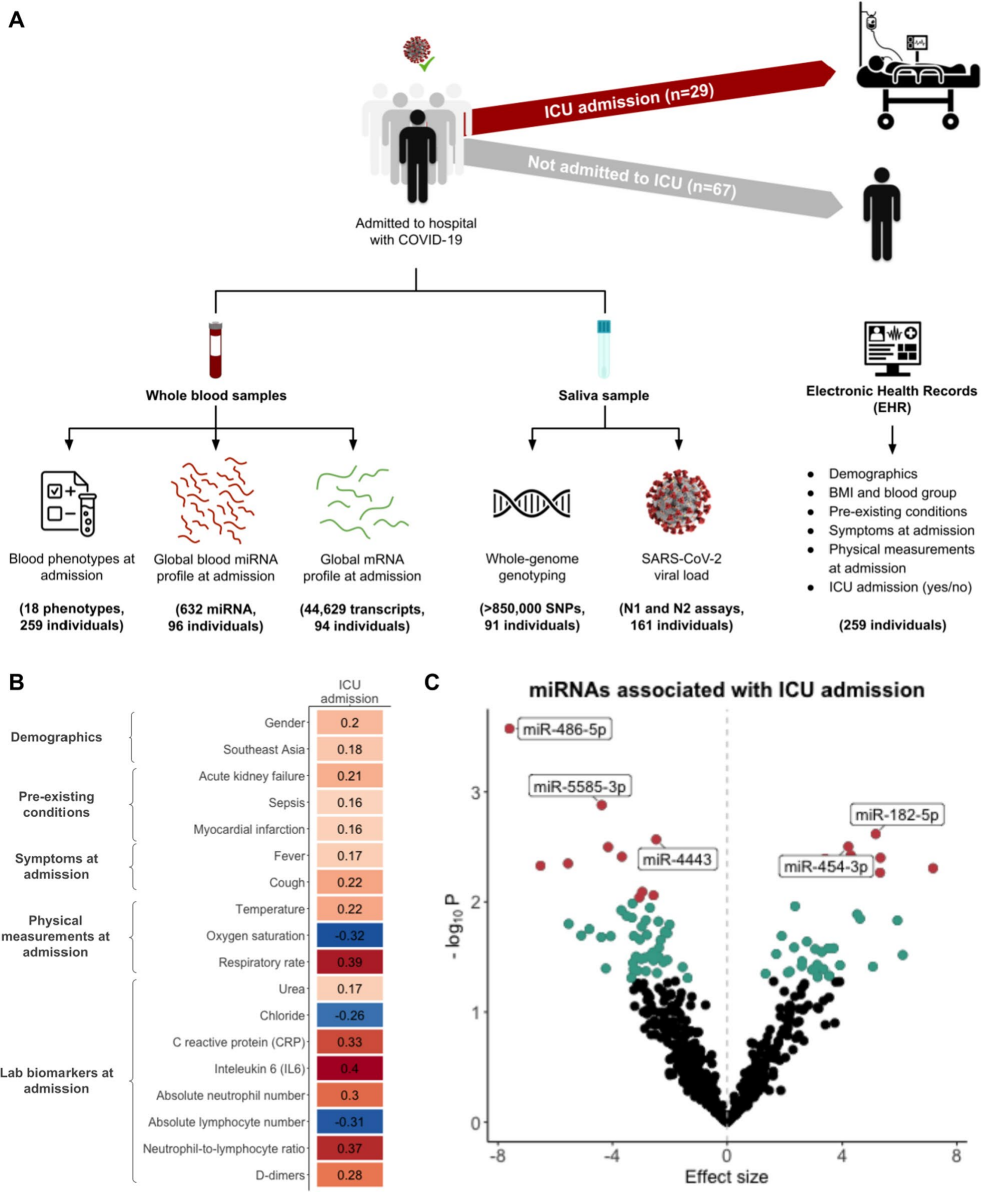
Clinical variables and miRNA levels at the time of hospital admission, prior to any clinical intervention or treatment, are associated with later ICU admission for COVID-19 patients. **A** Study design. **B** Significant Pearson correlations (FDR < 0.05) between 18 factors from electronic health records and ICU admission. **C** Volcano plots of miRNAs associated with ICU admission. Age and self-reported time from symptom onset to hospital admission were used as covariates in a logistic regression model. miRNAs significant at *p* < 0.05 are highlighted in blue, and miRNAs significant at *p* < 0.01 are highlighted in red, with the 5 most significant miRNAs labeled

To understand the clinical and biological factors underpinning COVID-19 disease severity, we analyzed electronic health records (EHRs) data for 259 unvaccinated patients and multi-omics data—genotypes, miRNA and RNA expression—for a subset of 96 patients (Fig. 1A). Among the 259 patients, 61 were admitted to the ICU (23.6%) at some point during their hospital stay; 65.3% of patients identified as male; the average age was 46.9 (SD = 14.2); and patients were predominantly from the Middle East and North Africa (MENA, 54.4%) and Southeast Asia (40.0%) (Additional file 2: Table S2A, see Additional file 1: Note 1 for classification of nationalities into regions). Around 75% of the cohort had at least one pre-existing condition, most commonly hypertension (47.1%) or diabetes (41.3%) (Additional file 2: Table S2A). The most common symptoms reported at the time of hospital admission were fever (54.4%) and cough (53.3%) (Additional file 2: Table S2B). Of the 259 patients, 96 were selected for miRNA and mRNA sequencing and genotyping (see Methods for selection criteria), including 29 patients (30.2%) that were admitted to the ICU. Due to technical reasons, RNA-seq data was not available for 2 of the 96 individuals. Notably, the distribution of demographics, pre-existing conditions and symptoms did not significantly differ between the full sample (*n* = 259) and the miRNA subset (*n* = 96) (Additional file 2: Table S2A).

### Several clinical variables and miRNA levels at the time of hospital admission are associated with later ICU admission

To identify factors associated with COVID-19 disease severity—using ICU admission as a proxy—we computed correlations between 62 variables from the EHR with ICU admission (see Additional file 1: Note 2 for a list of the 62 factors) in the full dataset of 259 individuals, and identified 18 significant correlations (FDR < 0.05). Being male (*r* = 0.20), from Southeast Asia (*r* = 0.18), and previously diagnosed with acute kidney failure (*r* = 0.21), sepsis (*r* = 0.16) or myocardial infarction (*r* = 0.16) were all positively correlated with ICU admission. At the time of hospital admission, self-reported symptoms of fever (*r* = 0.17) and cough (*r* = 0.22), as well as clinician-recorded body temperature (*r* = 0.22), oxygen saturation (*r* =  − 0.32) and respiratory rate (*r* = 0.39) were all correlated with later ICU admission. Notably, we identified 8 blood phenotypes significantly correlated with ICU admission, of which urea (*r* = 0.17), CRP (*r* = 0.33), IL-6 (*r* = 0.40), absolute neutrophil number (*r* = 0.30), NLR (*r* = 0.37) and D-dimers (*r* = 0.28) were positively correlated, while chloride (*r* = − 0.26) and absolute lymphocyte number (*r* = − 0.31) were negatively correlated (Fig. 1B; Additional file 2: Table S3A–B). Some of these associations are not independent, considering the correlation between some of the blood phenotypes (Additional file 1: Fig. S1). Interestingly, the viral load at time of hospital admission was not associated with later ICU admission in our dataset.

To understand whether miRNA levels at the time of hospital admission are associated with later ICU admission, we performed logistic regression analyses for each of the 632 miRNAs (see Methods for details on quality control of miRNA-seq data, Additional file 1: Fig. S2) in the sub-sample of 96 individuals, controlling for age and self-reported time from symptom onset to hospital admission (we did not control for gender because out of the 27 ICU patients, only 3 identified as women, Additional file 2: Table S3B). We identified 21 miRNAs whose levels at the time of hospital admission—10 downregulated, and 11 upregulated—were significantly associated (*p* < 0.01) with later ICU admission (Fig. 1C; Additional file 2: Table S4A; see results from a model adjusted for gender in Additional file 1: Fig. S4, Additional file 2: Table S4B). To understand the biological significance of the 21 ICU-associated miRNAs, we sought to identify their putative mRNA targets. After computing the Spearman correlations between the levels of the 21 miRNAs and 44,586 unique mRNA transcripts measured from the same blood sample collected at hospital admission (see Methods for details on quality control of RNA-seq data, Additional file 1: Fig. S4), we identified 15,336 correlated miRNA-mRNA pairs (FDR < 0.05), of which 6490 were negatively-correlated (mean *r* =  − 0.37, SD = 0.05), concerning 12 miRNAs (Additional file 1: Fig. S5A–B). Using IPA miRNA Target Prediction, we annotated the experimentally validated and/or highly predicted gene targets of the 12 miRNAs of interest (data was not available for 2 miRNAs), and found that 18 highly predicted miRNA-gene target pairs (6 miRNAs, 18 genes) were negatively correlated in our dataset (Additional file 2: Table S5; Additional file 1: Fig. S5C–H). These findings suggest that beyond clinical variables and blood phenotypes, miRNAs may also play a role in the host immune response to early COVID-19 infection. However, admission to the ICU is a complex phenotype resulting from both the patient’s clinical manifestation and the decision making of their clinical team. As such, ICU-associated miRNAs may not be most informative of underlying cellular mechanisms, which is why we next turned to study the miRNA architecture of the 8 ICU-associated blood endophenotypes.

### Numerous miRNAs are associated with ICU-associated phenotypes measured at the time of hospital admission

**Fig. 2:**
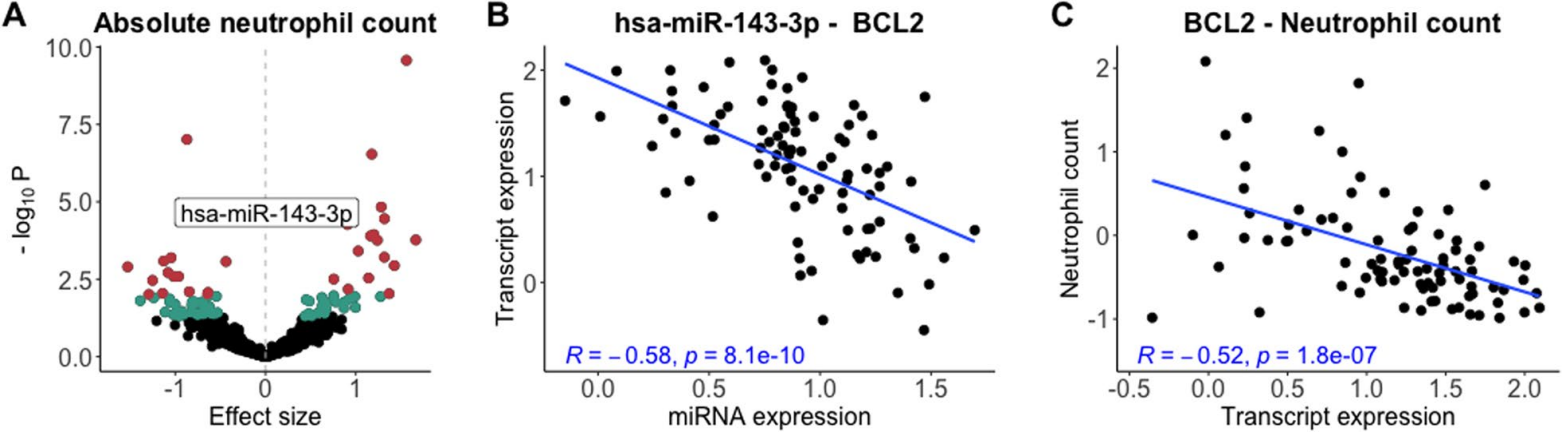
The positive association between hsa-miR-143-3p and neutrophil count is mediated by BCL2 expression. **A** Numerous miRNAs are associated with neutrophil count, including hsa-miR-143-3p (labeled). Both miRNA expression and blood phenotype levels were measured from the same blood sample, collected at the time of hospital admission. miRNAs significant at *p* < 0.05 are highlighted in blue. miRNAs significant at *p* < 0.01 are highlighted in red. Both miRNA expression and blood phenotype levels were standardized. **B** Correlation between hsa-miR-143-3p expression (*x*-axis) and BCL2 transcript expression (*y*-axis). **C** Correlation between BCL2 transcript expression (*x*-axis) and absolute neutrophil count (*y*-axis). The Pearson correlation and *p* value are in blue. miRNA expression, transcript expression and neutrophil count have all been standardized

To identify miRNAs associated with the 8 ICU-correlated blood endophenotypes, we performed linear regression analyses of the 632 miRNAs with each of the 8 blood endophenotypes, using standardized miRNA and blood phenotype levels, and adjusting for age and gender. We identified 9 miRNAs significantly associated with urea, 5 with chloride, 32 with CRP, 28 with neutrophil count (Fig. 2A), 20 with lymphocyte count, 18 with NLR, 16 with D-dimers (*p* < 0.01), and no miRNAs significantly associated with IL-6 (Additional file 1: Fig. S6; Additional file 2: Table S6A). We also quantified 5 of these miRNAs with qPCR, and found consistent associations between these miRNAs and their associated blood phenotypes in all 15 cases, of which 10 were also significant with qPCR-based data (Additional file 2: Table S6B). A total of 97 unique miRNAs were associated with at least one blood phenotype (for a comparison of results between models adjusted for age and gender, and models with no covariates, see Additional file 1: Fig. S7). In fact, we found that out of the 21 ICU-associated miRNAs, 5 were also associated with at least one blood phenotype: hsa-miR-4443, hsa-miR-450b-5p, p-hsa-miR-14, hsa-miR-150-3p, and hsa-miR-3615 (Additional file 1: Fig. S8), and noticed that many miRNAs were associated with more than one blood endophenotype, with neutrophil count and CRP sharing the maximum of 12 significant miRNA associations (Additional file 1: Fig. S9). These findings indicate that miRNAs may contribute to the complex biological mechanisms that regulate blood phenotypes during early stages of COVID-19 infection.

To understand the regulatory roles of the miRNAs associated with blood endophenotypes, we calculated Spearman correlations between the 97 miRNAs and 44,586 unique mRNA transcripts, and identified 50,427 significant negative correlations (FDR < 0.05), with a mean of − 0.37 (SD = 0.07), concerning 37 unique miRNAs (Additional file 1: Fig. S10). Using the IPA miRNA Target Prediction tool, we annotated the experimentally observed and highly predicted gene targets of the 37 miRNAs (data was not available for 9 miRNAs). We identified 16 experimentally observed miRNA-gene pairs that were negatively correlated in our dataset, corresponding to 16 genes targeted by 4 miRNAs—hsa-miR-21-5p, hsa-miR-338-5p, hsa-miR-199b-5p and hsa-miR-143-3p—most of which were associated with CRP, neutrophil count and NLR. We also observed 184 highly predicted miRNA-gene targets (20 miRNAs, 184 gene targets) that were negatively correlated in our dataset (Additional file 2: Table S7; Additional file 1: Fig. S11). Using IPA pathway enrichment analysis, we found that the 197 unique genes—pooled across experimentally observed and highly predicted gene targets—were implicated in MYC mediated apoptosis signaling, crosstalk between dendritic cells and natural killer cells, and p53 signaling, among others, and enriched in cancer, infectious disease and immunological disease (Additional file 2: Table S8A–B). Overall, these results imply that miRNAs associated with ICU-associated blood endophenotypes at the time of hospital admission putatively regulate genes involved in apoptotic and immunological pathways.

To test whether the effect of miRNAs on blood phenotypes is mediated by their regulation of gene expression, we performed medication analysis on the 599 unique triplets of miRNA, gene target and associated blood phenotype reported in Additional file 2: Table S7 (for genes with multiple transcripts, we only tested the transcript with the lowest *p* value). We identified 74 Bonferroni-significant mediations (*p* < 0.05), 8 of which concerned experimentally observed miRNA-gene target pairs. Most interestingly, we found that hsa-miR-143-3p—which is highly expressed in neutrophils (Juzenas et al., [42], Additional file 1: Fig. S12)—affects neutrophil count and NLR through the expression of BCL2, an apoptotic gene that regulates cell death (Fig. 2B, C; the negative correlation between hsa-miR-143-3p and BCL2 was also replicated in qPCR, *r* =  − 0.38, p = 2.4 × 10^−8^). We observed a few other notable examples: hsa-miR-199b-5p affecting NLR through the expression of ETS1, a transcription factor; hsa-miR-21-5p affecting neutrophil count and NLR through the expression of FASLG, an apoptosis-inducing transmembrane protein, as well as affecting NLR through the expression of TNF, a proinflammatory cytokine involved in cell proliferation, differentiation and apoptosis; and lastly, hsa-miR-338-5p influencing NLR by regulating BACE1, a protein involved in the proteolytic processing of the amyloid precursor protein (Additional file 2: Table S9). These patterns in our data provide further evidence for the role of miRNAs in regulating the expression of genes that are important for the host immune response to infection, and therefore, for response to SARS-CoV-2.

To further probe the relationship between miRNA expression and COVID-19 disease severity, we tested the association between miRNAs and ICU-associated clinical symptoms and self-reported symptoms at hospital admission (see Additional file 1: Note 2 for the full list of variables). We identified 2 miRNAs significantly associated with body temperature, 4 with oxygen saturation, and 16 with respiratory rate (*p* < 0.01) (Additional file 1: Fig. S13A–C; Additional file 2: Table S10A). Classifying individuals with 4 or more self-reported symptoms at hospital admission (out of 13) as highly symptomatic (note that the threshold of 4 symptoms was chosen because 4 is both the median and the mean of the number of symptoms reported), we found only 1 miRNA associated with being highly symptomatic (Additional file 1: Fig. S13D; Additional file 2: Table S10B).

### Some of the implicated miRNAs are genetically controlled by nearby genetic variants

**Fig. 3:**
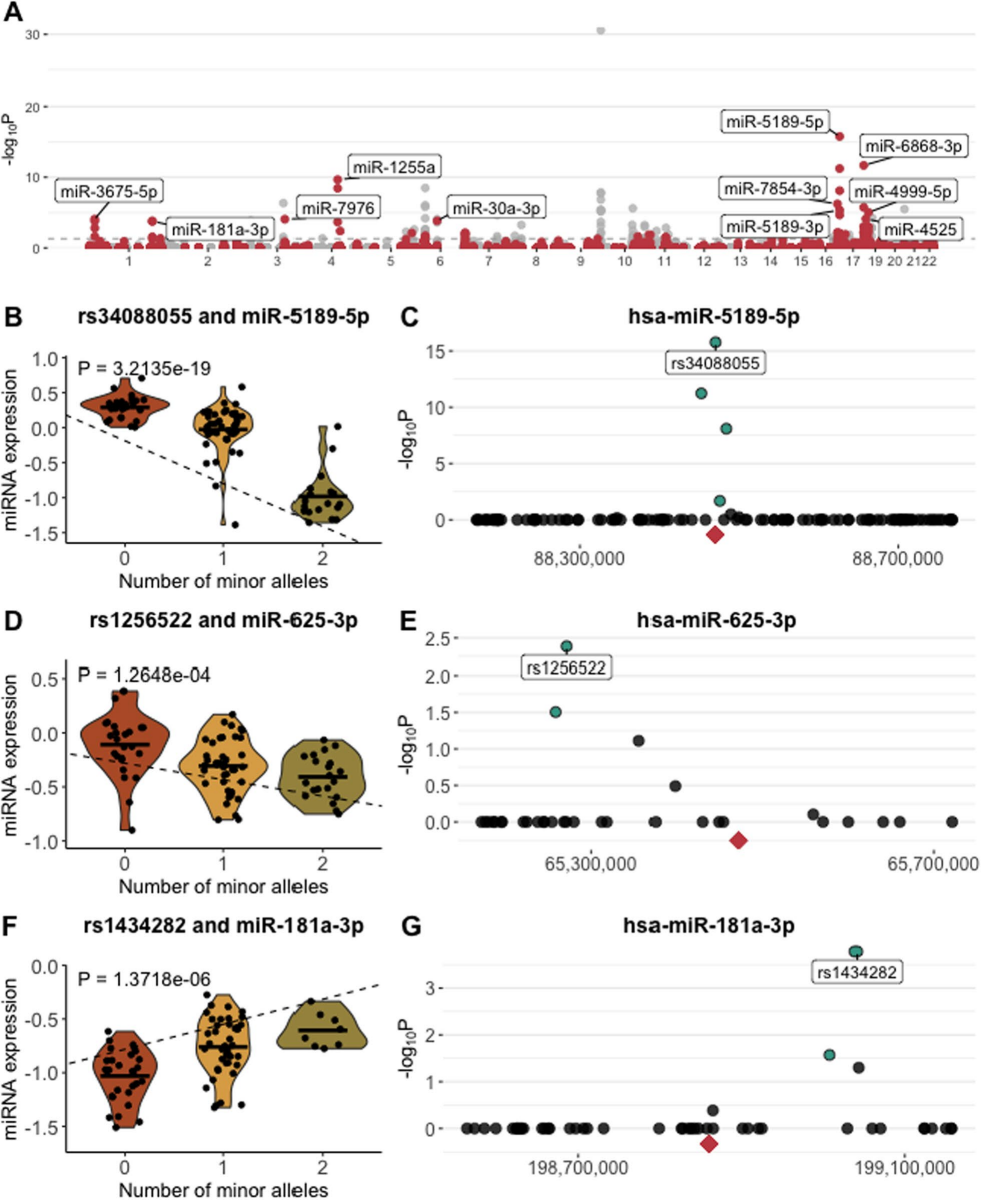
Expression of numerous miRNAs is genetically controlled by *cis*-eQTLs. **A** Manhattan plots showing all cis-eQTLs (defined as an association between a miRNA and a SNP in a 300,000 base pairs window). Points highlighted in pink show cis-eQTLs for miRNAs associated with one of the 8 blood phenotypes The dashed line corresponds to Bonferroni *p* < 0.05, and all points above the dashed line are significant cis-eQTLs. Labeled points refer to cis-eQTLs with Bonferroni *p* < 0.05, and some of the top cis-eQTLs are annotated with the e-miRNA. **B**–**G** Pairs of violin and fine-mapping plots for *cis-*eQTLs. The violin plot shows the linear relationships between the number of minor alleles and miRNA expression associated with each genotype. The dashed line corresponds to the linear regression fit, and the *p* value is stated on the plot. The fine-mapping plot shows all tested SNPs for each miRNA. Points highlighted in blue show e-SNPs significant at Bonferroni *p *< 0.05. Labeled point shows the top e-SNP for that cis-eQTL. The pink diamond shows the genomic position of the miRNA. **B**–**C** *cis*-eQTL hsa-miR-5189-5p and rs34088055. **E**–**F** *cis*-eQTL rs1256522 and hsa-miR-625-3p. **G**–**H** *cis*-eQTL rs1434282 and hsa-miR-181a-3p

Lastly, we tested whether genetic variation influenced the expression of miRNAs associated with the 8 blood endophenotypes. We performed *cis*-eQTL analysis using expression levels for 632 miRNAs and SNP data from 91 individuals (Additional file 1: Fig. S14; see Methods for details on quality control of genotyping data). For each miRNA, we tested between 1 and 671 SNPs, depending on the density of the SNP array within 300,000 base pairs (bp) from the miRNA (59 miRNAs were excluded from this analysis since there were no SNPs within this window). We identified a total of 168 significant *cis*-eQTLs (Bonferroni *p* < 0.05), of which 57 concerned 28 unique miRNAs that were associated with either ICU or an ICU-associated blood endophenotype (Additional file 2: Table S11). For each of the 28 miRNAs, we annotated the SNP with the lowest Bonferroni *P* value as the top SNP, and used wANNOVAR [43] to assign SNPs to genes, resulting in 28 top *cis*-eQTLs consisting of an e-SNP and an e-miRNA (Table 1, Fig. 3A). These *cis*-eQTLs reflect a significant linear relationship between the genotype (number of minor alleles) and miRNA expression levels (Fig. 3B, E, G), with 6 of the peak e-SNPs found within a 1000 bp window from the miRNA (Fig. 3D), and other e-SNPs found closely downstream (Fig. 3F) or upstream (Fig. 3H). In most scenarios, the top e-SNP was near other significant e-SNPs, likely due to linkage disequilibrium (e.g. Fig. 3F). The volcano plots and fine-mapping plots for other *cis*-eQTLs can be found in Additional file 1: Fig. S15. To test whether the effect of the e-SNP on the blood phenotype was mediated by e-miRNA expression, we performed mediation analysis for the 28 *cis*-eQTLs and their associated blood phenotypes, or for a total of 39 unique triplets of e-SNP, e-miRNA and blood phenotype. We identified 2 e-SNP-neutrophil associations that are mediated through miRNA expression (*p* < 0.00128, using a Bonferroni threshold): rs1256522 has effects on both neutrophil count and neutrophil/lymphocyte ratio that are mediated through the expression of hsa-miR-625-3p and rs79260648 has an effect on neutrophil/lymphocyte ratio mediated through the expression of hsa-miR-576-3p (Additional file 2: Table S12). Altogether, these results show that allelic variation influences the expression levels of miRNAs that contribute to the variation of ICU-associated blood endophenotypes during early stages of SARS-CoV-2 infection.

**Table 1:** List of top *cis*-eQTLs for 29 miRNAs associated with one of the 8 blood phenotypes correlated with ICU admission

SNP	miRNA	SNP position	A1	MAF	P Bonferroni	Distance SNP to miRNA (in BP)	Negative associations with ICU	Positive associations with ICU
rs34088055	hsa-miR-5189-5p	Chr16:88469262	T	0.44	1.70E-16	296	Neutrophil count	
rs7501512	hsa-miR-6868-3p	Chr17:76097225	A	0.41	2.10E-12	794	D-dimers	
rs1031034	hsa-miR-1255a	Chr4:101302229	A	0.29	2.18E-10	28,136	D-dimers	
rs2925983	hsa-miR-7854-3p	Chr16:81533035	C	0.38	5.96E-07	907	D-dimers	
rs12447180	hsa-miR-5189-3p	Chr16:88451314	C	0.45	4.42E-06	17,670	Neutrophil count, D-dimers	
rs28370965	hsa-miR-4999-5p	Chr19:8408107	T	0.22	9.60E-06	18,747	C-reactive protein	
rs9914549	hsa-miR-4525	Chr17:82667081	G	0.23	7.41E-05	1200	Lymphocyte count	
rs1765566	hsa-miR-3675-5p	Chr1:16708741	T	0.05	8.44E-05	150,247	Interleukin-6	
rs77318922	hsa-miR-7976	Chr3:127757054	C	0.07	8.64E-05	169,878	C-reactive protein	
rs9364148	hsa-miR-30a-3p	Chr6:71277249	A	0.07	0.000146	126,305	Urea	D-dimers
rs1434282	hsa-miR-181a-3p	Chr1:199041592	C	0.33	1.65E-04	182,502		D-dimers
rs11569501	hsa-miR-3940-3p	Chr19:6691620	A	0.08	1.03E-03	275,165	C-reactive protein	
rs76321536	hsa-miR-636	Chr17:76976432	G	0.12	0.001286	239,944	D-dimers	
rs79260648	hsa-miR-576-3p	Chr4:109205695	C	0.09	4.06E-03	283,057	Urea	Neutrophil count, Neutrophil-to-lymphocyte ratio
rs1256522	hsa-miR-625-3p	Chr14:65271311	C	0.44	0.004111	199,842		Neutrophil count, Neutrophil-to-lymphocyte ratio
rs1997243	hsa-miR-339-3p	Chr7:1044141	G	0.14	0.00711	21,164	C-reactive protein	
rs7710462	hsa-miR-5003-3p	Chr5:172643217	G	0.07	0.007797	19,005	C-reactive protein, Neutrophil-to-lymphocyte ratio	
rs60095937	hsa-miR-3177-3p	Chr16:1732963	A	0.05	0.009533	2075	D-dimers	
rs941449	hsa-miR-6777-3p	Chr17:17746517	C	0.2	0.01199	66,963	C-reactive protein, D-dimers, ICU admission	
rs4756822	hsa-miR-6073	Chr11:15695412	T	0.45	0.01375	274,143	C-reactive protein, D-dimers	
rs2495972	hsa-miR-1275	Chr6:33964665	G	0.49	0.0181	35,353		Lymphocyte count
rs11768761	hsa-miR-339-5p	Chr7:1030171	G	0.14	0.02617	7159		Urea
rs59088240	hsa-miR-342-5p	Chr14:99827961	A	0.06	0.02867	281,712	C-reactive protein, Neutrophil-to-lymphocyte ratio, D-dimers	
rs2382817	hsa-miR-6810-3p	Chr2:218286495	C	0.49	0.03112	55,463		Interleukin-6, D-dimers
rs9787810	hsa-miR-7155-5p	Chr11:64317826	T	0.24	0.0337	24,060	Neutrophil count, D-dimers	
rs1499294	hsa-miR-4742-3p	Chr1:224564806	C	0.32	0.04451	166,557	D-dimers	
rs62130995	hsa-miR-4746-5p	Chr19:4460286	A	0.13	0.0462	14,277	D-dimers, ICU admission	
rs4144630	hsa-miR-181a-2-3p	Chr9:124676921	T	0.36	0.04954	15,597		Chloride, Lymphocyte count

For each unique miRNA, the top SNP (lowest Bonferroni *P* value) is listed

## Discussion

In the three years since the emergence of SARS-CoV-2, scientific undertakings have improved our understanding of the host immune response to infection, and have led to the development of effective vaccines and improved treatments for COVID-19 disease. Yet the question of what contributes to the variation in COVID-19 disease severity—ranging from asymptomatic to severely symptomatic, and sometimes, lethal infection—remains standing. In this study, we pinpoint miRNA expression as a previously underappreciated mechanism for regulating blood phenotype levels during early stages of COVID-19, which, as correlated with later ICU admission, can be indicative of disease severity. We highlight 21 miRNAs whose levels at the time of hospital admission are associated with ICU admission, and 97 miRNAs associated with ICU-correlated blood endophenotypes, of which 5 are associated with both ICU admission and an endophenotype. Many of these miRNA have been reported as differentially expressed between COVID-19 patients and healthy controls (Additional file 1: Note 3, [34, 35, 39, 40, 44-47]). Through integrative miRNA-mRNA analysis, we identify 194 experimentally observed or highly predicted miRNA-gene target pairs that are negatively correlated in our dataset, and, using mediation analysis, find 8 instances where the miRNA affects neutrophil counts likely through transcriptional regulation of immune cell apoptosis. We furthermore describe the role of genetic variation in shaping miRNA expression levels—we characterize 28 top *cis*-eQTLs, and, using mediation analysis, document 3 examples of SNPs influencing neutrophil counts, mediated by miRNA expression.

Our results highlight two interesting aspects of the biology of SARS-CoV-2 infection that warrant further investigation. The first is the role of neutrophils in early response to SARS-CoV-2 infection. Not only were both neutrophil counts and neutrophil/lymphocyte ratio at the time of hospital admission correlated with later ICU admission, but also most of the miRNAs that negatively co-varied with their experimentally validated gene targets in our dataset were also associated with these two blood traits. We found that four of these miRNAs—hsa-miR-143-3p, hsa-miR-199b, hsa-miR-21-5p and hsa-miR-338-5p—affect these blood endophenotypes by regulating BCL2, ETS1, FASTLG, TNF and BACE1, many of which are involved in apoptotic (BCL2, FASTLG) and immune-related pathways (BCL2, TNF). These trends in our data are consistent with prior studies that have show enrichment of COVID-19-associated miRNAs in inflammatory and immune pathways [33] and highlighted neutrophils as key players in COVID-19 pathophysiology [15-17]. Secondly, some of the miRNAs highlighted in our study are strong candidates for functional follow-up. For instance, hsa-miR-143-3p—which affects neutrophil counts mediated by BCL2 expression—has been implicated in numerous immunological diseases such as cancers and ischemic stroke [48-50], has been found to influence inflammatory factors and cell apoptosis [51, 52], and to inhibit Wnt and MAPK signaling [53]. Similarly, hsa-miR-199b, which influences neutrophils through ETS1 expression, has previously been implicated in breast cancer [54, 55], and  described as a tumor suppressor in acute myeloid leukemia [56] and an inducer of apoptosis in oral cancer [51, 57]. Another promising candidate for functional follow-up is hsa-miR-21-5p, which affects neutrophils by regulating FASTLG and TNF expression, and has been previously implicated in colon, breast and gastric cancers [58-60].

This study has several strengths and some limitations. While previous studies have already pointed to genetic, miRNA and transcriptomic variation as predictive of COVID-19 disease severity, to our knowledge this is the first study to investigate the relationship between these three sources of biological variation in a matched, multi-omics dataset. This study design helped us prioritize miRNAs with miRNA-gene target correlation patterns that are consistent with a regulatory relationship, as well as identify miRNAs that are genetically controlled. More so, our study enrolled unvaccinated patients and investigated miRNA and RNA expression levels collected at the time of hospital admission, after a positive COVID-19 test, but before any medication or clinical intervention. By doing so, our results are free of numerous confounding factors, many of which are abundant in recent studies enrolling vaccinated patients. Lastly, our study cohort consists entirely of patients from MENA and South Asia, two geographical regions whose populations have been under-represented in COVID-19 research, as well as genetic research at large [61, 62]. The diversity of our study cohort (Additional file 1: Fig. S11) not only powered us to detect *cis*-eQTLs with large effects along a larger spectrum of genetic variation, but also to present findings that are potentially generalizable to around 30% of the world population living in MENA and South Asia. Another limitation of our study is the lack of matched controls, i.e. individuals uninfected with SARS-CoV-2. For this reason, we are unable to discern whether the miRNAs we report are induced only upon SARS-CoV-2 infection.

## Conclusion

In conclusion, this systems genetics study has given rise to the first genomic picture of the architecture of whole blood miRNAs in unvaccinated COVDI-19 patients. The results pinpoint post-transcriptional regulation as a potential mechanism that impacts blood traits underlying COVID-19 severity and warrant similar investigations in other populations. The study also reveals the association between host allelic genetic regulatory variation and miRNA expression levels in the context of COVID-19. Moreover, the multi-omics analysis presented demonstrated the value of the approach in capturing meaningful biological associations in cohorts of relatively small sample sizes.

## Methods

### Research ethics statement

The project was approved by the Research Ethics Committee at New York University Abu Dhabi (NYUAD) (HRPP-2020-59) and the Abu Dhabi COVID-19 Research International Review Board Committee of the Department of Health (Ref no: DOH/CVDC/2020/874).

### Study participants and enrollment

This was a prospective study of unvaccinated adult COVID-19 patients, where baseline sampling and phenotyping was done at the time of hospital admission following a positive COVID-19 test. Clinical follow-up was done during the course of the hospital stay. The study enrolled 264 patients across four hospitals in Abu Dhabi, UAE: Al Ain Hospital, Al Rahba Hospital, Mafraq Hospital and Sheikh Khalifa Medical Center, which are all managed under the same healthcare system authority, and therefore have the same procedures and clinical protocols. The recruitment period lasted from June to September 2020. Electronic consent forms were administered in English, Urdu, Hindi and Tagalog, depending on the participant’s preference; these four languages were selected as the most commonly spoken languages among patients in these clinics. The exclusion criteria included having Hb levels lower than 70 mmol/L, having platelet count less than 100,000/ml and/or having received transfusion within 24 h of potential study recruitment.

Of the 264 patients, 256 completed a questionnaire upon enrollment which asked about the date of symptom onset and the experienced symptoms. Participants also provided biological specimens (whole blood samples for genotyping and miRNA/RNA extraction, and saliva for viral load quantification) at the time of hospital admission. Sample collection was done following unified procedures, and samples were randomized throughout downstream experiments to avoid potential batch effects. At the end of the study recruitment period, electronic health records (EHRs) were extracted for 259 patients (from hereafter referred to as the “study cohort”), which included information about demographics, pre-existing conditions and an extensive documentation of their COVID-19-related hospital stay, including physical measurements and lab tests. All clinical and biological data was de-identified. All analyses including correlations between EHR variables—for example, correlations between blood phenotypes and ICU admission—were conducted in the full sample of 259 patients.

### RNA extraction

In the clinic, blood was collected in Tempus tubes and refrigerated at 4 °C before being transported to the research labs at NYU Abu Dhabi (NYUAD). Whole blood RNA was isolated using the Tempus Spin RNA Isolation Kit (Thermo Fisher) following manufacturer’s instructions. Quantification and quality control of the extracted RNA was performed using a 2100 Bioanalyzer instrument and a Qubit 2.0 Fluorometer.

### Selection of the miRNA subsample

miRNA sequencing was performed on 96 patient blood samples collected at the time of hospital admission, prior to any clinical interventions or treatments. We focused on 96 patients from the full set of 259 participants, maximizing the number of ICU patients from MENA and South Asia—populations that are underrepresented in existing COVID-19 studies and well-represented in our study cohort—and individuals with complete clinical data. To maximize matching between patients admitted to the ICU and those who were not, we kept all patients who identified as women (since they comprised only 35% of the full sample), and all patients who identified as men and were admitted to the ICU; note that within the EHR in the UAE, sex is reported as either a “man” or a “woman”. Finally, we prioritized patients with complete relevant clinical data in their EHR. All analyses including miRNA and RNA expression were conducted in this sub-sample of 96 patients.

### miRNA sequencing

Small RNA libraries were prepared from 400 ng of high-quality total RNA (RNA Integrity Number RIN > 8) using the NEBNext Multiplex Small RNA Library Prep Set for Illumina (New England Biolabs). Size selection of small RNA cDNA libraries was done using the gel purification method. The library size distribution was checked using a 2100 Bioanalyzer instrument to ensure correct size amplicons are selected for sequencing. All samples and libraries were randomized and processed in the same way to minimize batch effects. Individual libraries were quantified, and equimolar quantities of each library were pooled and sequenced on an S1 flow cell using a NovaSeq instrument (Illumina). The miRNA data is deposited in GEO under accession GSE220077.

### Bioinformatic analysis of miRNA data

Raw miRNA sequencing reads were demultiplexed and converted to FASTQ files using the standard Illumina pipeline with bcl2fastq. Sequences were processed with Trimmomatic v0.36 to remove indexes and adapter sequences. Trimmed reads were processed with the FASTX Toolkit v0.0.14 to filter out reads with tail quality < 15 nucleotides and retain reads of 16–25 nucleotides for downstream analyses. FastQC v0.11.5 was used to visualize quality metrics before and after the filtering. High-quality reads were subject to small RNA annotation and quantification using OASIS [63, 64]. miRNAs with a minimum count of 5 reads in at least 50% of the samples were retained, log2 transformed and standardized (mean = 0, SD = 1) (Additional file 1: Fig. S2). For each miRNA, individuals who were outliers for miRNA expression—having miRNAs expression values more/less than + 3/-3 standard deviations from the mean—were removed.

### Replication of miRNA expression with qPCR

The expression of five miRNAs (hsa-miR-21-5p, assay ID: 000397; hsa-miR-143-3p, assay ID: 002249; hsa-miR-150-3p, assay ID: 002637; hsa-miR-625-3p, assay ID: 002432, and hsa-miR-5189-3p, assay ID: 466901_mat, all from Thermo) were validated using quantitative PCR (qPCR). Total RNA was extracted from 92 samples (leaving 4 spaces in a 96-well-plate for negative controls), which were the same as those used for miRNA-sequencing, and re-quantified using the Qubit RNA Broad Range Kit (Thermo Fisher Scientific). Of those, 88 samples passed initial quality control. Next, 20 ng of RNA from each sample was reverse transcribed using the TaqMan MicroRNA Reverse Transcription Kit following the manufacturer's instructions. After cDNA synthesis, miRNAs were pre-amplified using a mixture of 1 µL of PreAmp master mix (Standard Biotools), 1.25 µL of pooled TaqMan miRNA Assays (0.2X), 1.5 µL of water, and 1.25 µL of cDNA. The pre-amplification reaction was cycled under the following conditions: 95 °C for 2 min, followed by 14 cycles of 95 °C for 15 s and 60 °C for 4 min, and finally held at 4 °C. The pre-amplified reactions (5 µL) were then diluted in a 96-well plate with 20 µL of low TE buffer (Thermo Fisher Scientific). Finally, qPCR of the miRNAs was performed using the Gene Expression with the 192.24 IFC using Fast TaqMan Assays protocol (PN 100-6174 C1, Standard Biotools) on the Juno and Biomark HD instruments for sample and assay loading and qPCR, respectively.

### RNA sequencing

RNA sequencing was performed on RNA extracted from the whole blood of the 96 patients with miRNA data generated. The extracted RNA samples (400 ng) were subject to globin and ribosomal RNA depletion using the NEBNext® Globin & rRNA Depletion Kit (as per manufacturer’s protocol; New England Biolabs). Preparation of cDNA libraries was subsequently performed using the NEBNext® Ultra II Library Prep Kit for Illumina (New England Biolabs). Libraries were checked for quality and quantified with a 2100 Bioanalyzer instrument, and then pooled into one lane of an S2 flow cell and 101-bp paired-end sequenced on a NovaSeq instrument (Illumina) in XP mode. The mRNA data is deposited in GEO under accession GSE220076.

### Bioinformatic analysis of RNA-seq data

Raw reads were processed for quality control: first, using Trimmomatic v0.36 to remove adapter sequences and low-quality bases (using the parameters ILLUMINACLIP: trimmomatic_adapter.fa:2:30:10 TRAILING:3 LEADING:3 SLIDINGWINDOW:4:15 MINLEN:36), and then, using the Fastp program to remove sequencing artifacts and poly-G tails. Filtered reads were then mapped to the human reference genome (Ensembl GRCh38.p4 release-81) using HISAT v2.0.4 with default options other than --dta. The resulting SAM output was converted to sorted BAM using SAMtools v1.3.1. Raw count per gene was calculated from the sorted bam for individual samples using the options (-s no -t exon -I gene_id) in Htseq-count program. Transcript abundance quantification was performed using Stringtie v1.3.0, and raw gene counts from Htseq-count were converted to TPM (transcripts per million) using COEX-Seq R shiny app program. Transcripts with a minimum of 1 TPM in 50% of the samples were retained for downstream analyses, resulting in 44,629 unique transcripts that mapped to 15,545 unique genes. The RNA-seq data was then log10 transformed and standardized (mean = 0, SD = 1) (Additional file 1: Fig. S4). Following the same pipeline for miRNA filtering, for each transcript, we removed individuals who were outliers for transcript expression.

### Viral load quantification

Automated extraction of viral RNA from 300 μL of patients’ saliva was performed using the Chemagic 360 automated nucleic acid extraction system (2024-0020, Perkin Elmer, Waltham, MA, USA) and the Chemagic Viral DNA/RNA 300 Kit H96 (CMG-1033S, Perkin Elmer, Waltham, USA) according to the manufacturer’s instructions. RNA was eluted in 80 μL elution buffer followed by reverse transcription (RT), preamplification and quantitative PCR (qPCR) using the Fluidigm Real-Time PCR for Viral RNA Detection protocol (FLDM-00103, Fluidigm, San Francisco, CA, USA).

Viral load was quantified in saliva samples from 161 COVID-19 patients using a microfluidic ultra-sensitive quantitative test [65]. Per CDC recommendations, two assays were used for SARS-CoV-2 detection: 2019-nCoV_N1 and 2019-nCoV-N2 (2019-nCoV CDC EUA Kit, 10006606, IDT). The human RNase P (RP) assay was used as a control for RNA extraction and in RT-qPCR reactions. Each sample was analyzed using 9 replicates for N1, 9 replicates for N2, and 6 replicates for RP assays. Details of the quantitative nature of the microfluidic test are described in detail in [65]. The Ct values were converted to copies/µL using standard curves based on 100-fold serial dilutions of Twist RNA and SARS-CoV-2 plasmids ranging from 5 to 50,000 copies/µL. Viral load was calculated as the mean viral load from the N1 and N2 assays, given the high concordance of the N1 and N2 assays (Pearson *r* = 0.91). The viral load data was log2 transformed and standardized.

### Genotyping

Whole-genome genotyping was performed for the 96 samples selected for miRNA/RNA sequencing using the UAE Healthy Future Study [66] custom design Axiom genotyping array which contains > 850,000 single-nucleotide polymorphisms (SNPs) with 90% similar content to the Axiom PMDA array (ThermoFisher). 400 μl of whole blood samples were used for genomic DNA extraction with the Chemagic DNA isolation kit. DNA quantification and quality check were carried out using NanoDrop spectrophotometer followed by gel electrophoresis for DNA integrity check. Total genomic DNA (200 ng) was amplified and randomly fragmented into 25 to 125 base pair (bp) fragments. These fragments were purified, re-suspended, and hybridized to the Axiom arrays. Following hybridization, the bound target was washed under stringent conditions to remove nonspecific background caused by random ligation events. Each polymorphic nucleotide was queried via a multi-color ligation event carried out on the array surface. After ligation, the arrays were stained and imaged on the GeneTitanTM Multi-Channel Instrument, and a row intensity data file (.CEL file) was generated for each sample.

### Genotyping calling and quality control

The row intensity files were analyzed using Applied Biosystems Axiom™ Analysis Suite software, which automates data analysis and includes allele-calling algorithms. Following the best practice genotyping analysis workflow, samples were filtered for dish QC ≥ 0.82, QC call rate ≥ 97, and average call rate ≥ 98.5. SNPs with a call rate cutoff ≥ 95 and Fisher’s linear discriminant cutoff ≥ 3.6 were retained.

### Bioinformatic analysis of genotyping data

Genotyping data was filtered using PLINK [67] to remove SNPs with minor allele frequency (MAF) < 5%, Hardy Weinberg Equilibrium (HWE) test *p* value < 0.005, genotype missingness > 10% and individual missingness > 10%, resulting in 91 individuals and 263,838 SNPs. *Cis*-eQTL analyses were performed using a linear model adjusted for age and gender. For each of the 632 miRNA and 661 unique genetic positions, SNPs within 300,000 base pairs from the middle genomic position of the miRNA were tested (note that for miRNAs with more than one genetic position due to copy number variation, each *cis* region were tested independently), and *p* values were adjusted using Bonferroni correction [68]. The following PLINK commands were used: --bfile --no-pheno --allow-no-sex --chr --from-kb --to-kb --pheno --covar --covar-name --linear hide-covar --adjust --out. Principal component analysis (PCA) was performed with PLINK using genotyping data from this study (*n* = 96) merged with genotyping data from 2504 individuals of the 1000 Genomes Project (1000 [69] based on 238,313 common SNPs.

### Statistical analysis

All statistical analysis and data visualization were performed using R statistical software v. 4.0.2. Results were reported as significant if the nominal *p* value < 0.01, or an adjusted *p* value (FDR or Bonferroni) < 0.05 depending on the analysis as detailed in the results section. In figures and tables, statistical significance was reported using the following criteria: ns (*p* > 0.05), * (*p* < 0.05), ** (*p* < 0.01), *** (*p* < 0.001), and **** (*p* < 0.0001), except in the mediation analyses where Bonferroni-corrected *p* value threshold was used and reported as * (*p* < *P*_Bonferonni_), ns (*p* > *P*_Bonferonni_).

Associations between miRNAs levels at admission and ICU admission were calculated using logistic regression models adjusted for age and the time from COVID-19 symptom onset to hospital admission (the self-reported time from symptom onset to hospital admission meant to control for the fact that hospital admission occurred at a different stage of the COVID-19 disease for different patients). Associations between miRNAs and continuous blood phenotypes, both measured at admission and from the same blood sample, were calculated using linear regression models adjusted for age and gender. For continuous variables, outliers—defined as having values + / − 3 SD from the mean—were removed, and then variables were standardized (mean = 0, SD = 1). Causal mediation analysis was performed using the mediate() function in R. The fitted models for the mediator and the outcome were linear. Results include the average causal mediation effects (ACME), the ACME confidence interval, and bootstrapped *p* values.

## Supplementary Information

**Additional file 1.** Supplementary figures, and supplementary note.

**Additional file 2.** Supplementary Tables detailing patient characteristics, miRNAs associated with ICU and ICU-associated phenotypes, and detected cis-eQTLs.

## Data Availability

The miRNA data is deposited in GEO under accession GSE220077, and the mRNA data is deposited in GEO under accession GSE220076. All code and phenotype data used for analyses is available at https://github.com/Yidaghdour/covid-miRNA.
