# Supplementary material for: Systems genetics identifies miRNA-mediated regulation of host response in COVID-19
Source: Hum Genomics. 2023 Jun 12;17:49. doi: 10.1186/s40246-023-00494-4 (PMC10257974; doi:10.1186/s40246-023-00494-4)
Supplement: Supplementary file 1 — Additional file 1. Supplementary figures, and supplementary note. [file 40246_2023_494_MOESM1_ESM.docx]

**SUPPLEMENTARY FIGURES**


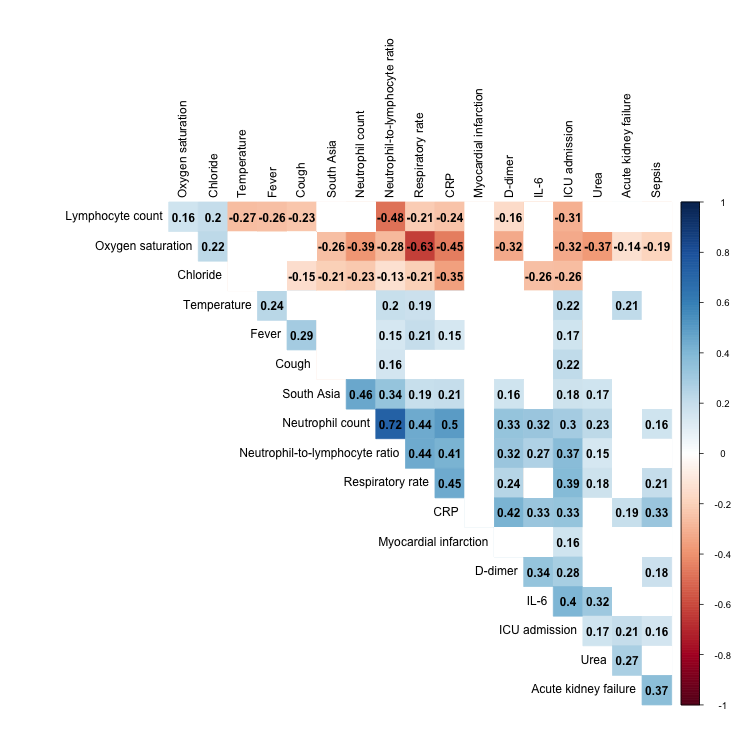


**Supplementary Figure 1.** Correlogram of ICU admission and the 18 clinical variables significantly associated with it. Plot only shows significant Pearson correlations (FDR < 0.05).

**
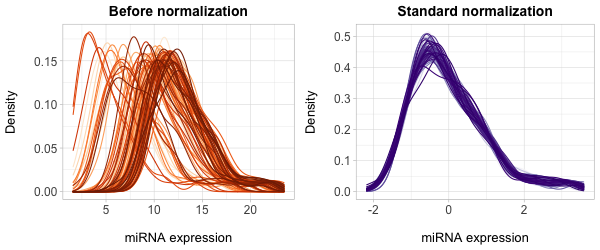
**

**Supplementary Figure 2.** Density plots of microRNA expression before and after standardization. Each line represents one of the 96 individuals in the dataset.

**
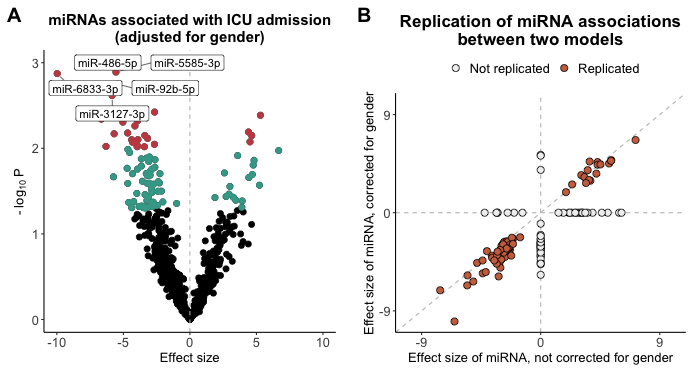
**

**Supplementary Figure 3.** (A) Volcano plot of miRNAs associated with ICU admission. Age and self-reported time from symptom onset to hospital admission were used as covariates in a logistic regression model. miRNAs significant at p < 0.05 are highlighted in blue. miRNAs significant at p < 0.01 are highlighted in red. The 5 most significant miRNAs are labeled. (B) Comparison of effect sizes of miRNA associations with ICU admission, with gender as a covariate (x-axis), and without gender as a covariate (y-axis). Age and self-reported time from symptom onset were used as covariates in both models.

**
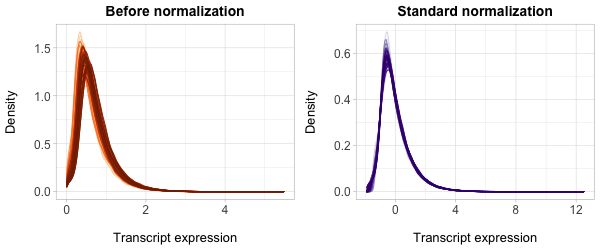
**

**Supplementary Figure 4.** Density plots of transcript expression before and after standardization. Each line represents one of 94 individuals in the dataset.

**
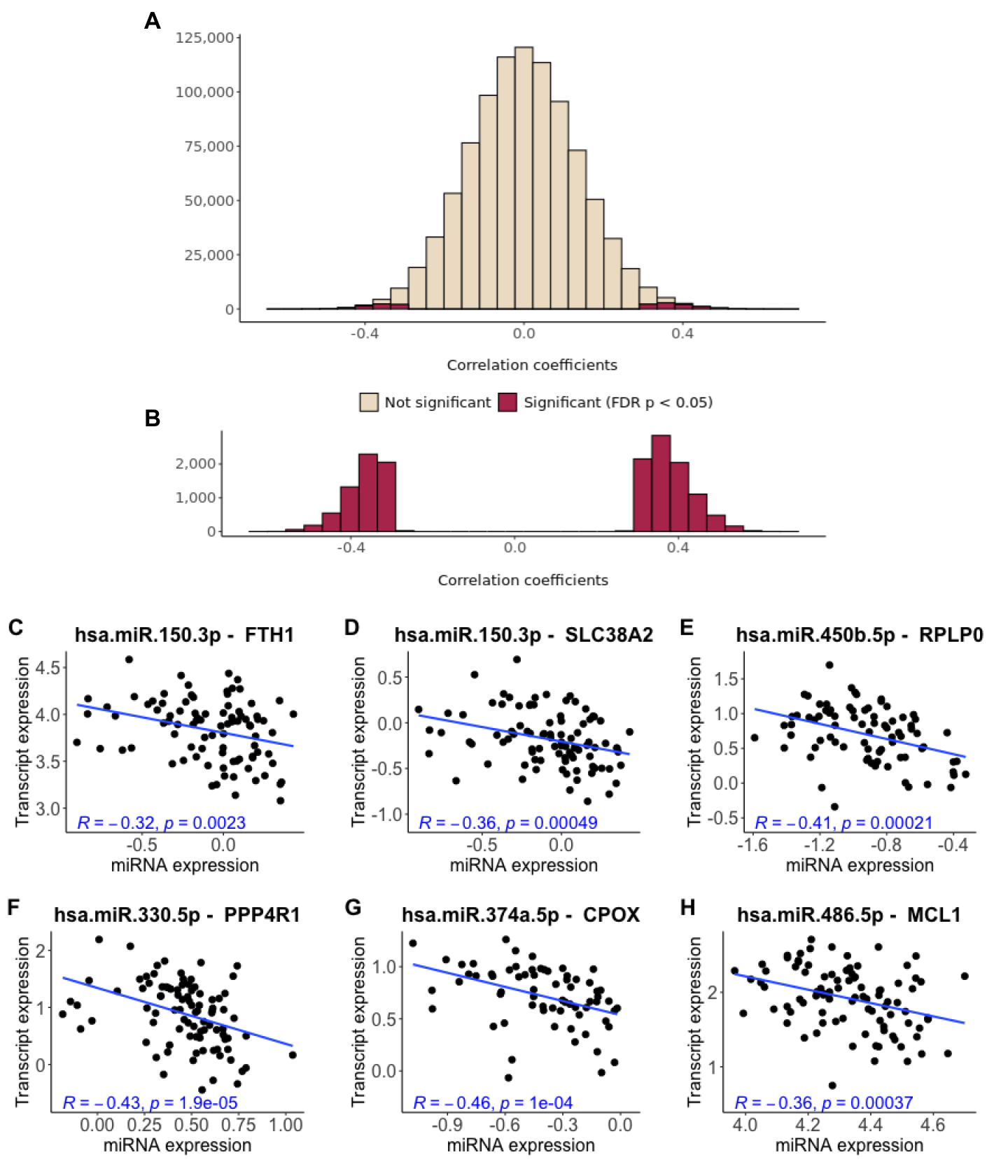
Supplementary Figure 5.** (A-B) Distribution of miRNA-mRNA Spearman correlation coefficients for the 21 miRNAs significantly associated with ICU admission for all correlations (A), and significant correlations (B). (C-H) Examples of highly predicted miRNA-gene target pairs whose levels were negatively correlated in our dataset. Both the miRNA expression (x-axis) and the transcript expression (y-axis) were standardized.

**
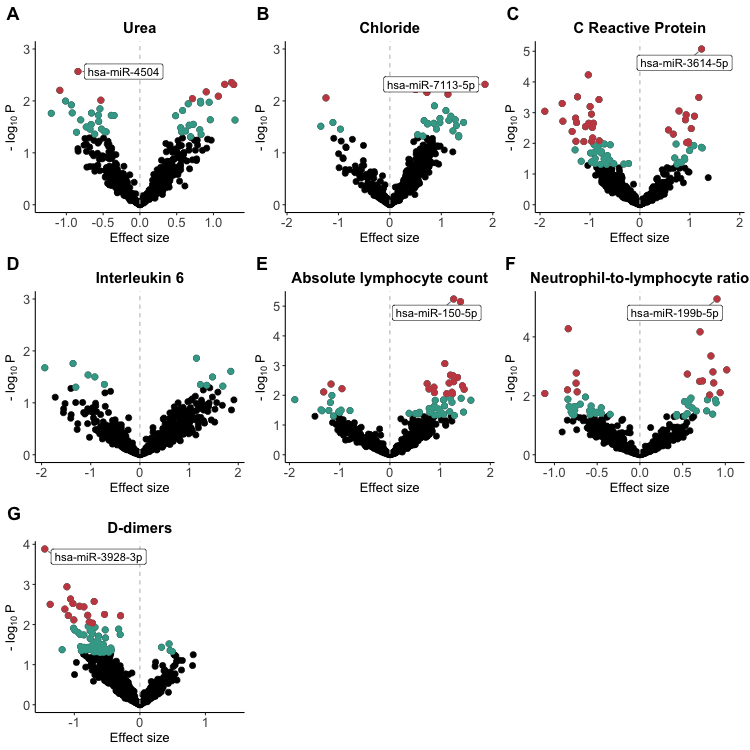
**

**Supplementary Figure 6. Numerous miRNAs are associated with each of the 8 blood phenotypes correlated with ICU admission.** Both miRNA expression and blood phenotype levels were measured from the same blood sample, collected at the time of hospital admission. miRNAs significant at p < 0.05 are highlighted in blue. miRNAs significant at p < 0.01 are highlighted in red. The top miRNA for each blood phenotype (lowest P-value, p < 0.01) is labeled. Both miRNA expression and blood phenotype levels were standardized.

**
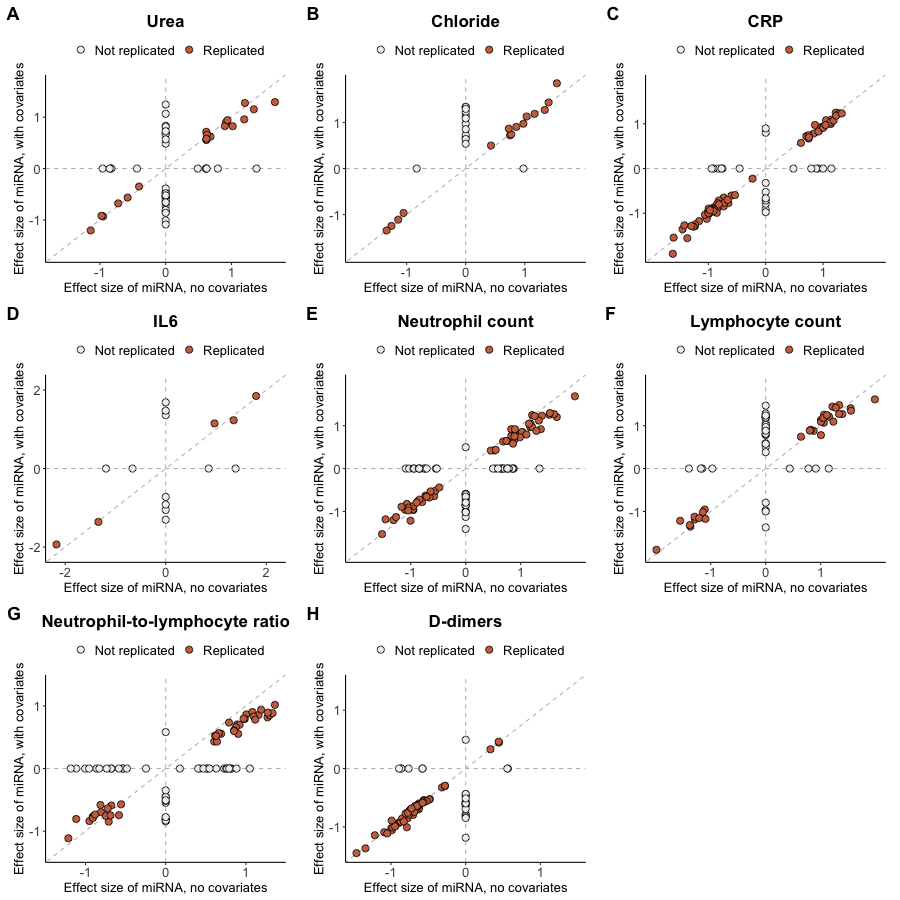
**

**Supplementary Figure 7.** Comparison of effect sizes of miRNA associations with different blood phenotypes, in unadjusted models (no covariates, x-axis) and adjusted models (age and gender as covariates, y-axis).

**
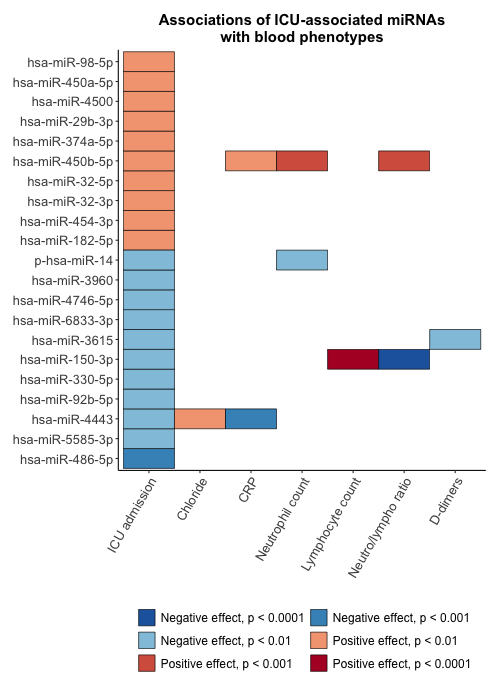
**

**Supplementary Figure 8.** Some of the miRNAs associated with ICU admission are also associated with blood phenotypes.


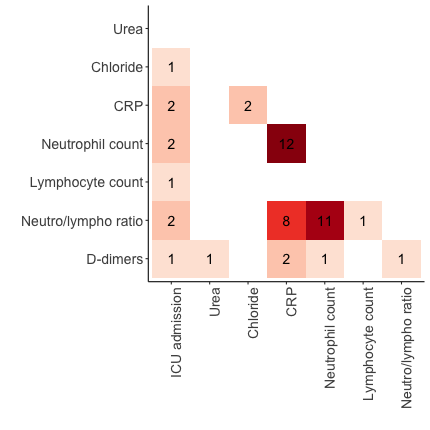


**Supplementary Figure 9.** Number of miRNA associations shared between pairs of blood phenotypes. Blank tile means that the two phenotypes share no significant (p < 0.01) miRNAs associations.


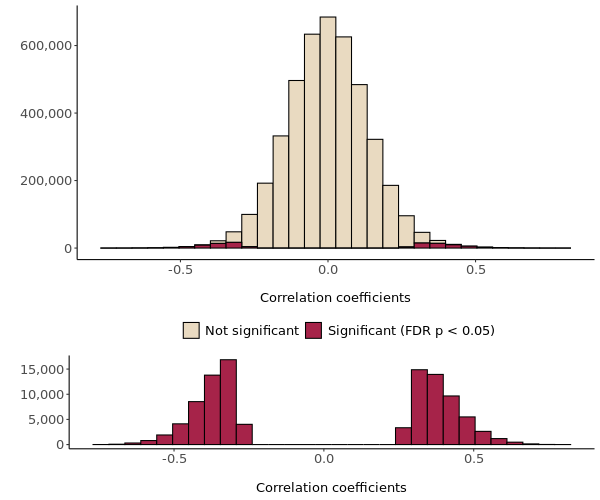


**Supplementary Figure 10.** (A-B) Distribution of miRNA-mRNA Spearman correlation coefficients for the 97 miRNAs significantly associated with ICU-associated blood phenotypes for all correlations (A), and significant correlations (B).

**
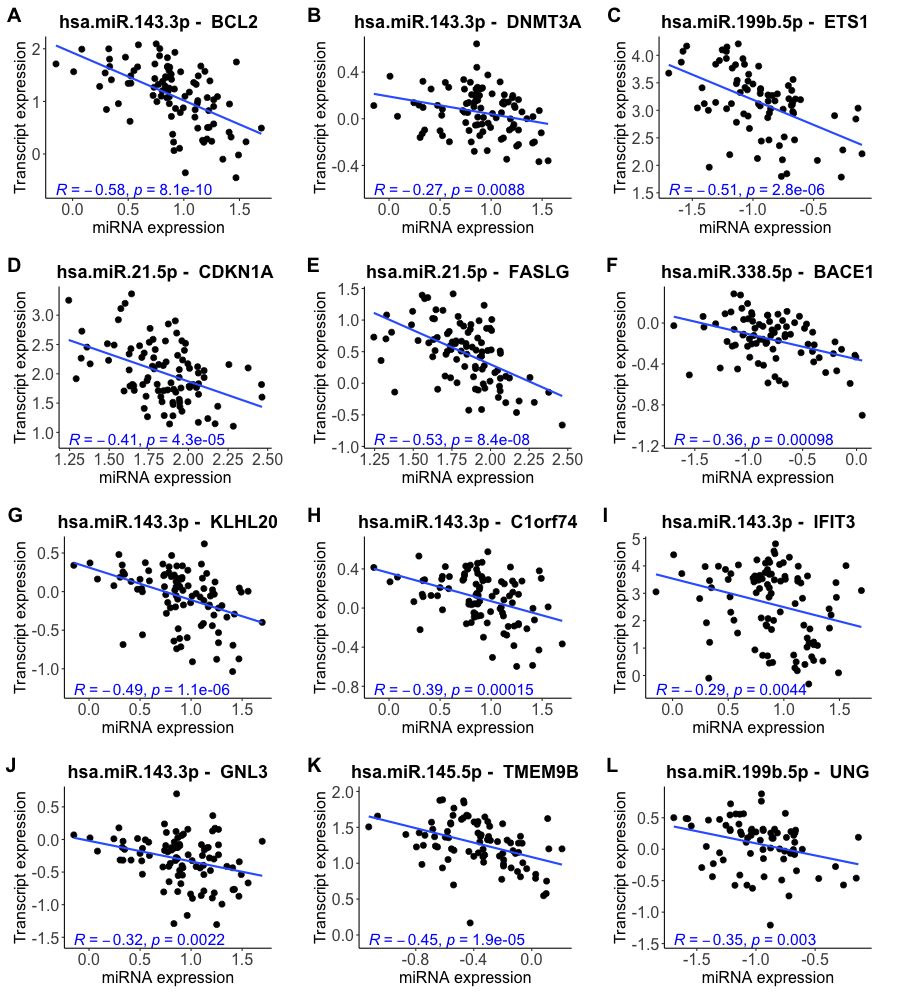
**

**Supplementary Figure 11.** Examples of miRNA-mRNA cross correlations for miRNAs associated with one of the 8 blood phenotypes. (A-F) Experimentally observed miRNA-gene target pairs that were negatively correlated in our dataset. (G-L) Highly predicted miRNA-gene target pairs that were negatively correlated in our dataset. Both the miRNA expression (x-axis) and the transcript expression (y-axis) have been standardized.

**
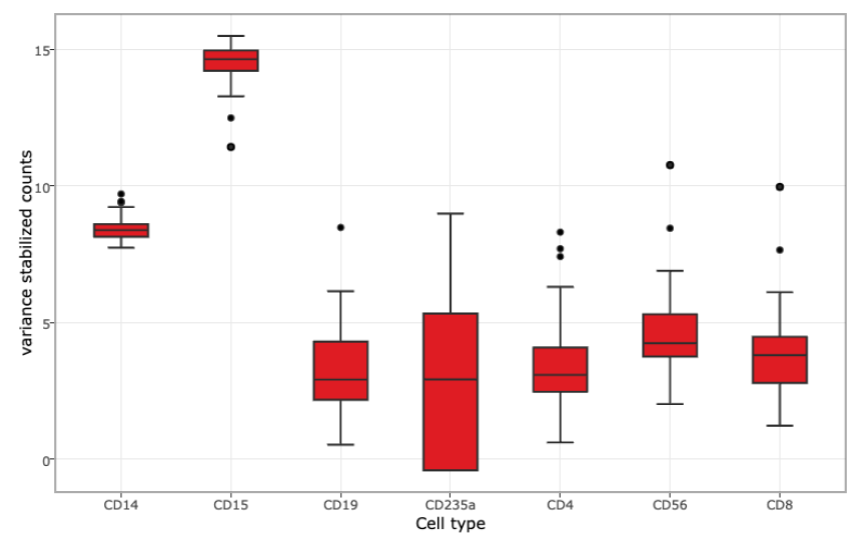
**

**Supplementary Figure 12. Expression of hsa-miR-143-3p in different human peripheral blood types.** Figure adapted from Juzenas et al. 2017, and screenshotted from <http://134.245.63.235/ikmb-tools/bloodmiRs/>.


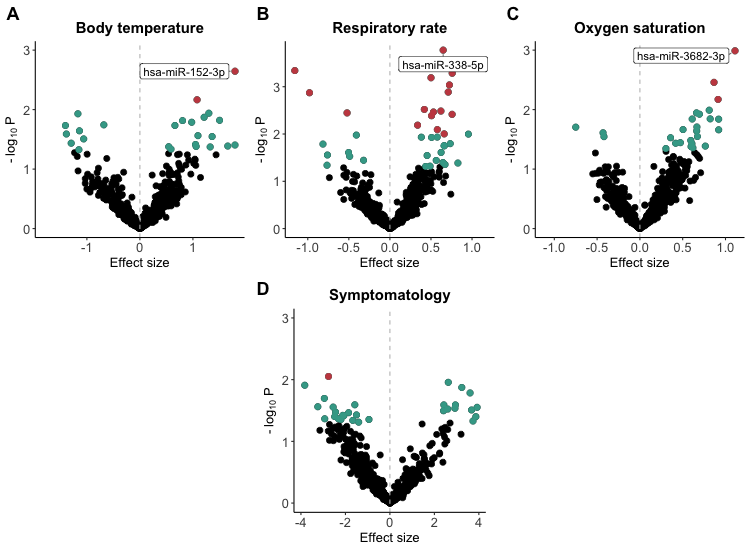


**Supplementary Figure 13. Numerous miRNAs are associated with clinical and self-reported proxies of COVID-19 disease severity.** (A-C) Clinical proxies of severity: temperature, respiratory rate, and oxygen saturation. (D) Being highly symptomatic is a self-reported proxy of severity; it refers to self-reporting 4 or more symptoms (out of 13) at time of hospital admission. Both miRNA expression and physical measurements were measured from the same blood sample, collected at the time of hospital admission. Both miRNA expression and physical measurements were standardized. miRNAs significant at p < 0.05 are highlighted in blue. miRNAs significant at p < 0.01 are highlighted in red. The top miRNA for each blood phenotype (lowest P-value, p < 0.01) is labeled.


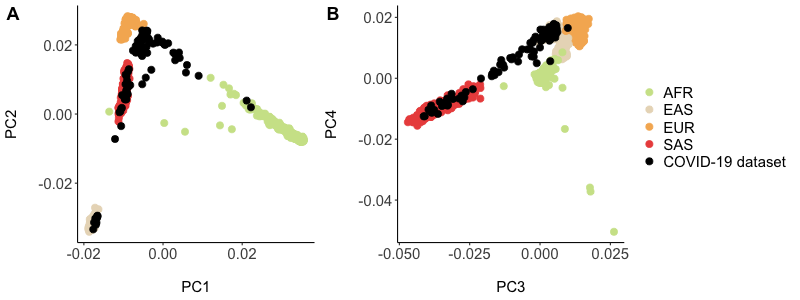


**Supplementary Figure 14.** Principal component analysis (PCA) of the COVID-19 dataset (this study) and four reference super-populations from the 1000 Genome Project.


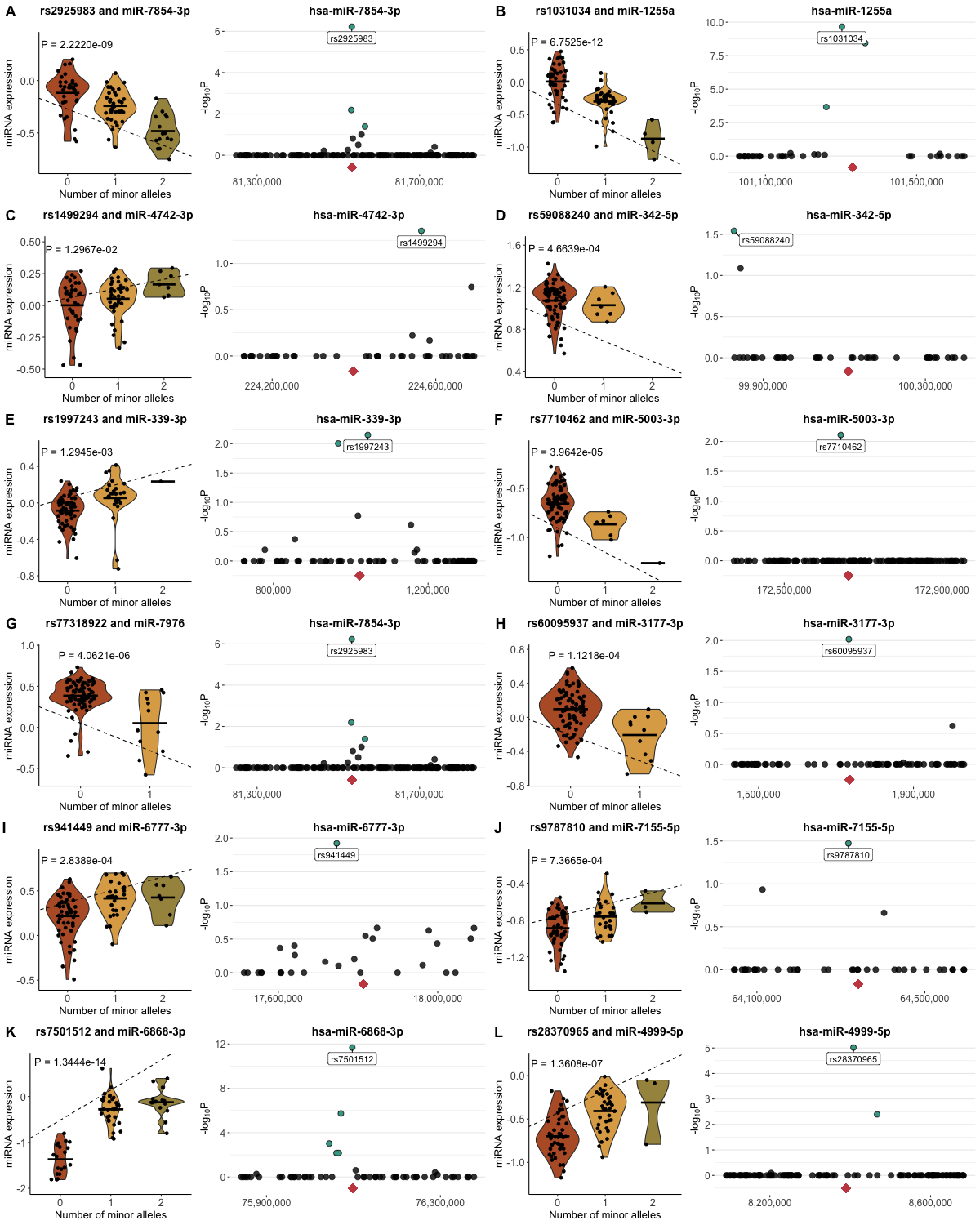


**Supplementary Figure 15.** Pairs of violin and fine-mapping plots for *cis-*eQTLs not included in Figure 3. The violin plot shows the linear relationships between the number of minor alleles and miRNA expression associated with each genotype. The dashed line corresponds to the linear regression fit, and the p-value is stated on the plot. The fine-mapping plot shows all tested SNPs for each miRNA. Points highlighted in blue show e-SNPs significant at Bonferroni P < 0.05. Labeled point shows the top e-SNP for that cis-eQTL. The pink diamond shows the genomic position of the miRNA.

**SUPPLEMENTARY NOTES**

1. **Classifications by regions**

The patients in the study reported 25 different nationalities, which we had categorized into 4 large regions as follows:

| **Region** | **Nationalities** |
| --- | --- |
| Middle East and North Africa (MENA) | Egyptian, Emirati, Iranian, Iraqi, Jordanian, Lebanese, Moroccan, Omani, Palestinian, Syrian, Tunisian, Yemeni |
| Africa (excluding MENA) | Comoran, Ethiopian, Sudanese, Ugandan |
| Southeast Asia | Bangladeshi, Filipino, Indian, Indonesian, Nepalese, Pakistani, Sri Lankan |
| North America and Australia | American, Australian |

1. **List of potential COVID-19 factors**

This is the full list of COVID-19 factors that were collected and tested for correlations with ICU admission:

| **Category** | **Independent variables** |
| --- | --- |
| Demographics | - Age - Gender - MENA - Southeast Asia - Africa (excluding MENA) - North America and Australia |
| Physical measurements | - BMI - Temperature - Oxygen saturation - Respiratory rate - Systolic pressure - Diastolic pressure |
| Blood groups | - Blood group A - Blood group B - Blood group AB - Blood group O - Blood group - positive - Blood group - negative |
| Pre-existing conditions | - Hypertension - Diabetes - Asthma - Ischemic heart disease - Cancer - Acute kidney failure - Chronic kidney disease - Fungal infection - HIV - Sepsis - Myocardial infarction - Number of existing conditions |
| Symptoms at admission | - Fever - Fatigue - Headache - Cough - Sore throat - Difficulty breathing - Runny nose - Muscle pain - Nausea - Loss of appetite - Loss of sense of taste - Loss of sense of smell - Eye pain |
| Lab tests | - Urea - Chloride - CO2 - Potassium - Magnesium - Phosphorous - Bilirubin - C reactive protein (CRP) - Troponin T - Vitamin D OH - Interleukin 6 (IL6) - Absolute neutrophil number - Absolute lymphocyte number - Neutrophil-to-lymphocyte ratio - HbA1c - Procalcitonin - D-dimers - HEPB |
| Other | - SARS-CoV-2 viral load |

1. **Comparison of miRNA results to other studies**
2. Li et al., 2021b identified 6 miRNAs that are differentially expressed between COVID-19 patients and healthy controls. Of these, **3 (50%)** were associated with ICU or an ICU-associated blood phenotype in our dataset:

| **miRNA** | **Association in our dataset** |
| --- | --- |
| miR-6501-5p | Lymphocyte count |
| miR-183-5p | Interleukin-6 |
| miR-144-3p | ICU admission |

1. Gutmann et al., 2022 identified 69 miRNAs that were significantly differentially expressed between healthy controls and COVID-19 patients with severe disease. Of these, **31 (45%)** were associated with ICU or an ICU-associated blood phenotype in our dataset.

| **miRNA** | **Association in our dataset** |
| --- | --- |
| miR-4454 | Interleukin-6 |
| miR-3960 | Chloride, C-reactive protein, Neutrophil count |
| miR-199b-5p | C-reactive protein, Neutrophil count, Neutrophil-to-lymphocyte ratio |
| miR-210-3p | C-reactive protein |
| miR-21-5p | C-reactive protein, Neutrophil count, Neutrophil-to-lymphocyte ratio |
| miR-22-3p | Chloride |
| miR-15a-5p | Chloride |
| miR-32-5p | C-reactive protein, Neutrophil count, D-dimers |
| miR-505-3p | Neutrophil count, Neutrophil-to-lymphocyte ratio |
| miR-199a-5p | Neutrophil count, Neutrophil-to-lymphocyte ratio |
| miR-324-3p | Urea |
| miR-425-5p | Lymphocyte count |
| miR-92a-3p | ICU admission |
| miR-93-3p | Urea, Neutrophil count |
| miR-181a-3p | D-dimers |
| miR-98-5p | Lymphocyte count |
| miR-26b-5p | Lymphocyte count |
| miR-181d-5p | C-reactive protein, Neutrophil count |
| miR-146b-5p | Chloride, Lymphocyte count |
| miR-let-7a-5p | Urea |
| miR-146a-5p | Lymphocyte count |
| miR-363-3p | Lymphocyte count |
| miR-10a-5p | D-dimers |
| miR-342-3p | C-reactive protein, Lymphocyte count, Neutrophil-to-lymphocyte ratio |
| miR-183-5p | Interleukin-6 |
| miR-145-3p | C-reactive protein, Neutrophil count, Neutrophil-to-lymphocyte ratio |
| miR-182-5p | ICU admission |
| miR-let-7f-5p | Lymphocyte count |
| miR-4508 | C-reactive protein, Neutrophil count |
| miR-181a-2-3p | Chloride, Lymphocyte count |
| miR-150-5p | Lymphocyte count |

1. Farr et al., 2021 identified 42 miRNAs that were significantly differentially expressed between healthy controls and COVID-19 patients (that did not receive oxygen treatment). Of these, **15 (35.7%)** were associated with ICU or an ICU-associated blood phenotype in our dataset.

| **miRNA** | **Association in our dataset** |
| --- | --- |
| hsa-miR-99b-3p | Urea, Neutrophil count, Neutrophil-to-lymphocyte ratio |
| hsa-miR-1273h-3p | C-reactive protein, Neutrophil count, Neutrophil-to-lymphocyte ratio |
| hsa-miR-576-5p | Chloride |
| hsa-miR-6772-3p | C-reactive protein |
| hsa-miR-874-3p | Lymphocyte count |
| hsa-miR-92a-3p | ICU admission |
| hsa-miR-651-5p | C-reactive protein |
| hsa-miR-1468-5p | Chloride |
| hsa-miR-345-5p | C-reactive protein |
| hsa-miR-339-3p | C-reactive protein |
| hsa-miR-6503-3p | Interleukin-6 |
| hsa-miR-132-3p | D-dimers |
| hsa-miR-877-5p | D-dimers |
| hsa-miR-450b-5p | C-reactive protein, Neutrophil count, Neutrophil-to-lymphocyte ratio |
| hsa-miR-30a-3p | Urea, D-dimers |

1. Fernández-Pato et al., 2022 identified 200 miRNAs that were differentially expressed between SARS-CoV-2 infected individuals and healthy controls. Of these, **55 (27.5%)** were also associated with ICU or an ICU-associated blood phenotype in our dataset.

| **miRNA** | **Association in our dataset** |
| --- | --- |
| hsa-miR-4665-5p | ICU admission |
| hsa-miR-331-3p | Urea, C-reactive protein |
| hsa-miR-4525 | Lymphocyte count |
| hsa-miR-6786-3p | ICU admission |
| hsa-miR-5010-5p | Interleukin-6 |
| hsa-miR-6868-3p | D-dimers |
| hsa-miR-6804-5p | C-reactive protein |
| hsa-miR-6511a-3p | ICU admission |
| hsa-miR-3180 | Neutrophil count, D-dimers |
| hsa-miR-98-3p | Urea, D-dimers |
| hsa-miR-671-5p | C-reactive protein, Neutrophil count |
| hsa-miR-581 | Urea |
| hsa-miR-1273h-5p | D-dimers |
| hsa-miR-197-5p | Lymphocyte count |
| hsa-miR-7854-3p | D-dimers |
| hsa-miR-3605-5p | ICU admission |
| hsa-miR-5100 | Chloride, C-reactive protein |
| hsa-miR-3614-5p | C-reactive protein, Neutrophil-to-lymphocyte ratio |
| hsa-miR-27b-5p | Lymphocyte count |
| hsa-miR-629-5p | C-reactive protein, Neutrophil count, Neutrophil-to-lymphocyte ratio, D-dimers |
| hsa-miR-4443 | Chloride, C-reactive protein, Neutrophil count |
| hsa-miR-760 | C-reactive protein |
| hsa-miR-651-5p | C-reactive protein |
| hsa-miR-15a-5p | Chloride |
| hsa-miR-99b-3p | Urea, Neutrophil count, Neutrophil-to-lymphocyte ratio |
| hsa-miR-22-3p | Chloride |
| hsa-miR-125b-5p | Lymphocyte count |
| hsa-miR-576-3p | Urea, Neutrophil count, Neutrophil-to-lymphocyte ratio |
| hsa-miR-1273h-3p | C-reactive protein, Neutrophil count, Neutrophil-to-lymphocyte ratio |
| hsa-miR-5010-3p | D-dimers |
| hsa-miR-1246 | Neutrophil count, Lymphocyte count |
| hsa-miR-501-3p | Urea, C-reactive protein |
| hsa-miR-25-5p | Lymphocyte count, Lymphocyte count |
| hsa-miR-99b-5p | C-reactive protein, Neutrophil count, Neutrophil count, Neutrophil-to-lymphocyte ratio |
| hsa-miR-27b-3p | Neutrophil-to-lymphocyte ratio |
| hsa-miR-7-5p | Neutrophil count, Lymphocyte count, D-dimers |
| hsa-miR-21-5p | C-reactive protein, Neutrophil count, Neutrophil-to-lymphocyte ratio |
| hsa-miR-501-5p | Urea, Lymphocyte count |
| hsa-miR-93-3p | Urea, Neutrophil count |
| hsa-miR-6805-5p | C-reactive protein |
| hsa-miR-139-5p | Neutrophil-to-lymphocyte ratio |
| hsa-miR-671-3p | Urea |
| hsa-miR-155-5p | Neutrophil count, Lymphocyte count, D-dimers |
| hsa-miR-4746-5p | D-dimers |
| hsa-miR-30b-5p | Urea |
| hsa-miR-342-3p | C-reactive protein, Lymphocyte count, Neutrophil-to-lymphocyte ratio |
| hsa-miR-146b-5p | Chloride, Lymphocyte count |
| hsa-miR-7848-3p | C-reactive protein, Neutrophil count, Neutrophil-to-lymphocyte ratio |
| hsa-miR-132-5p | D-dimers |
| hsa-miR-150-3p | C-reactive protein, Neutrophil count, Lymphocyte count, Neutrophil-to-lymphocyte ratio, D-dimers |
| hsa-miR-342-5p | C-reactive protein, Neutrophil-to-lymphocyte ratio, D-dimers |
| hsa-miR-150-5p | Lymphocyte count |
| hsa-miR-3158-3p | D-dimers |
| hsa-miR-659-5p | Urea |
| hsa-miR-1275 | Lymphocyte count |

1. Chen et al., 2021 identified 32 miRNAs that were differentially expressed between SARS-CoV-2 infected individuals and healthy controls. Of these, **9 (28.1%)** were also associated with ICU or an ICU-associated blood phenotype in our dataset.

| **miRNA** | **Association in our dataset** |
| --- | --- |
| hsa-miR-3614-5p | C-reactive protein, Neutrophil-to-lymphocyte ratio |
| hsa-miR-652-3p | Urea |
| hsa-miR-192-5p | Neutrophil count, Lymphocyte count |
| hsa-miR-581 | Urea |
| hsa-miR-1291 | Neutrophil count |
| hsa-miR-3615 | D-dimers |
| hsa-miR-659-5p | Urea |
| hsa-miR-132-5p | D-dimers |
| hsa-miR-4473 | D-dimers |

1. Li et. al., 2021b identified 23 miRNAs that were differentially expressed between SARS-CoV-2 infected individuals and healthy controls. Of these, **8 (34.8%)** were also associated with ICU or an ICU-associated blood phenotype in our dataset.

| **miRNA** | **Association in our dataset** |
| --- | --- |
| hsa-miR-144-3p | ICU admission |
| hsa-miR-96-5p | C-reactive protein, Lymphocyte count |
| hsa-miR-183-5p | Interleukin-6 |
| hsa-miR-21-5p | C-reactive protein, Neutrophil count, Neutrophil-to-lymphocyte ratio |
| hsa-miR-18a-5p | ICU admission |
| hsa-miR-125b-5p | Lymphocyte count |
| hsa-miR-6501-5p | Lymphocyte count |
| hsa-miR-32-5p | C-reactive protein, Neutrophil count, D-dimers |

1. Kim et. al., 2021 identified 5 miRNAs that were predicted to commonly bind to SARS-CoV, MERS-CoV, and SARS-CoV-2, and that showed differential expression patterns in hamster lungs before and after infection with SARS-CoV-2. Of these, **1 (20%)** was also associated with ICU-associated phenotypes in our dataset.

| **miRNA** | **Association in our dataset** |
| --- | --- |
| hsa-miR-15b-5p | Lymphocyte count, Neutrophil-to-lymphocyte ratio |

1. Latini et al., 2022 highlighted the functional impact of hsa-let-7b-5p on modulating ACE2 and DPP4 levels. They found that hsa-let-7b-5p was downregulated in naso-oropharyngeal swabs of COVID-19 patients compared to controls. In our dataset, we found that **hsa-let-7b-5p** was associated with urea levels, and hsa-let-7b-**3p** was associated with ICU admission.
